# Plexin-A1 is required for Toll-like receptor-mediated microglial activation in the development of lipopolysaccharide-induced encephalopathy

**DOI:** 10.3892/ijmm.2014.1690

**Published:** 2014-03-07

**Authors:** TAKUJI ITO, KENJI YOSHIDA, TAKAYUKI NEGISHI, MASAYASU MIYAJIMA, HYOTA TAKAMATSU, HITOSHI KIKUTANI, ATSUSHI KUMANOGOH, KAZUNORI YUKAWA

**Affiliations:** 1 Department of Physiology, Faculty of Pharmacy, Meijo University, Nagoya 468-8503, Japan; 2 Laboratory Animal Center, Wakayama Medical University, Wakayama 641-8509, Japan; 3 Department of Immunopathology, Research Institute for Microbial Diseases, Osaka University, Osaka 565-0871, Japan; 4 Department of Molecular Immunology, Research Institute for Microbial Diseases, Osaka University, Osaka 565-0871, Japan

**Keywords:** semaphorin, plexin, microglia, lipopolysaccharide

## Abstract

Recent investigations have suggested that semaphorins, which are known repulsive axon guidance molecules, may play a crucial role in maintaining brain homeostasis by regulating microglial activity. Sema3A, secreted in higher amounts from injured neurons, is considered to suppress excessive inflammatory responses by inducing microglial apoptosis through its binding to Plexin-A1 receptors on activated microglia. To clarify the *in vivo* role of Plexin-A1-mediated signaling in lipopolysaccharide (LPS)-induced injury in mouse brain, we examined the neuroinflammatory changes initiated by LPS administration to the cerebral ventricles of wild-type (WT) and Plexin-A1-deficient (−/−) mice. WT mice administered LPS exhibited a significantly higher expression of COX-2, iNOS, IL-1β and TNF-α in the hippocampus, and a significantly greater ventricular enlargement and intracerebral infiltration of leukocytes, as compared with the saline-treated group. By contrast, Plexin-A1−/− mice administered LPS did not exhibit a significantly increased expression of COX-2, iNOS, IL-1β or TNF-α in the hippocampus as compared with the saline-treated group. Plexin-A1−/− mice administered LPS did not show significant increases in ventricle size or infiltration of leukocytes into the brain, as compared with the saline-treated group. In WT, but not in the Plexin-A1−/− primary microglia treated with LPS, Sema3A induced significantly more nitric oxide production than in the immunoglobulin G control. These results revealed the crucial role of the Sema3A-Plexin-A1 interaction in the Toll-like receptor 4-mediated signaling of the LPS-induced activation of microglia. Thus, results of the present study revealed the essential role of Plexin-A1 in the development of LPS-induced neuroinflammation in mice, suggesting the possible application of microglial control of the semaphorin-plexin signaling system to the treatment of LPS-induced encephalopathy and other psychiatric diseases associated with neuroinflammation.

## Introduction

Microglia, which are a crucial component of the innate immune system of the central nervous system (CNS), are known to play crucial roles in regulating neuroinflammation (1). Activated microglia are known to perform beneficial functions in defense and tissue repair in the CNS (1). Findings of previous studies have shown that activated microglia also propagate inflammation in the CNS through antigen presentation and production of proinflammatory cytokines or chemokines, reactive oxygen species, and nitric oxide (NO) (2–4). For example, Toll-like receptor 4 (TLR4), a member of the Toll-like receptor family, is involved as a lipopolysaccharide (LPS) receptor in the activation of microglia (5). Activation of TLR4 expressed in microglia leads to neuronal injury through its ligation with LPS (6). Blockade of microglial activity suppresses the development of inflammatory lesions in an experimental autoimmune encephalitis model (7). However, the manner in which immunoregulatory molecules controlling microglial activity are involved in the development of neuroinflammation remains to be determined.

Semaphorins and their receptors exhibit various functions in axon guidance, organogenesis, angiogenesis, tumorigenesis, and immune regulation (8–18). The primary receptors for semaphorins are known to be members of the plexin family (9,19–21). Plexin-A1, in combination with ligand-binding neuropilins, transmits repulsive axon guidance signals for soluble class III semaphorins inside the axonal growth cone (22). Plexin-A1 has also been shown to play important roles in the developmental stages of chick heart by working as a receptor for the transmembrane semaphorin, Sema6D, in a neuropilin-independent manner (23). Furthermore, Plexin-A1 expressed in dendritic cells is involved in T-dendritic cell interactions in the immune system (24).

Sema3A, a ligand of the Neuropilin-1-Plexin-A1 coreceptor complex also plays a role as an inducer of neuronal apoptosis during the embryonic stage (25). However, recent identification of Sema3A expression in injured adult brain suggests that semaphorins affect neural regeneration (26,27). Upregulation of Sema3A mRNA and Neuropilin-1 and -2 mRNA after middle cerebral artery occlusion suggested that reciprocal contact between injured neurons and activated microglia may promote phagocytosis of damaged neurons (26). The expression of Plexin-A1 in rat microglia has also been identified, suggesting that Sema3A produced by injured neurons suppresses neuroinflammation by inducing apoptosis of activated microglia through binding to the Neuropilin-1-Plexin-A1 coreceptor complex (28). As described above, neuroinflammation may be induced extensively in Plexin-A1-deficient (Plexin-A1−/−) brain by abnormally activated microglia, since apoptosis hardly occurs in Plexin-A1−/− microglia. Therefore, microglial responses to inflammatory stimuli may be more intensified in Plexin-A1−/− brain than in wild-type (WT) brain. To explore this possibility, we compared the level of neuroinflammation induced by intracerebroventricular (ICV) administration of LPS to WT and to Plexin-A1−/− mice. Contrary to our prediction, LPS-induced neuroinflammation was significantly weaker in Plexin-A1−/− mice than in WT mice. Thus, the present findings indicated a mechanism in which Plexin-A1 expressed in microglia is integral to the optimal production of inflammation-related mediators, such as cytokines, following TLR4 stimulation.

## Materials and methods

### Animals

Plexin-A1−/− mice were generated by gene targeting with E14.1 embryonic stem (ES) cells (29). Briefly, the gene targeting vector was designed to replace the genomic region containing the initiation codon and the Sema domain-coding sequence with a neomycin-resistance gene, and then transfected into E14.1 ES cells by electroporation. G418- and ganciclovir-resistant clones were screened by polymerase chain reaction (PCR) and confirmed by Southern blotting. Mutant ES cells were introduced into mouse blastocysts and then transferred into pseudopregnant mice to generate chimeras. F1 heterozygous knockout mice were generated by breeding the chimeras with Balb/c mice and were then backcrossed with Balb/c mice for 10 generations. Pairs of resultant heterozygous mice were bred to gain homozygous knockout mice and their WT littermates as controls.

The mice were housed in the Wakayama Medical University animal facilities and the animal center in the Faculty of Pharmacy of Meijo University. The care and use of mice as well as other experimental protocols were conducted in accordance with the guidelines promulgated by the Physiological Society of Japan and the guidelines on animal experimentation of both Wakayama Medical University and Meijo University. The Animal Ethics Review Committee of both institutions approved the experimental protocol.

### Genotype analysis

Genotyping was performed by PCR with mouse tail DNA as the template and a Plexin-A1 gene-specific primer set as previously reported (29).

### Isolation of primary microglia and immunocytochemistry

For optimal dissociation of tissue samples, brain tissue of WT and Plexin-A1−/− pups from postnatal day 1–3 (P1–3) was dissociated using a Neural Tissue Dissociation Kit (Sumitomo Bakelite Co., Ltd., Tokyo, Japan). Microglia were isolated from the single-cell suspension by MACS Technology using CD11b MicroBeads (Miltenyi Biotec, Bergisch Gladbach, Germany) according to the manufacturer’s instructions. The isolated microglia were then cultured for 24 h in a culture medium (Sumitomo Bakelite). The cells were then fixed in 4% paraformaldehyde in phosphate-buffered saline (PBS) and processed for immunocytochemistry. After fixed cultures were permeabilized with 0.2% Triton X-100 in PBS for 5 min, the microglia were stained with isolectin IB4 conjugated with Alexa 488 (Life Technologies, Carlsbad, CA, USA) for microglial identification and visualization with anti-Plexin-A1 antibodies (Abcam, Cambridge, MA, USA). Alexa 594-conjugated secondary antibodies were used to visualize primary antibody staining, and DAPI was added for the final 20 min of incubation for nuclear identification.

### Reverse transcription (RT)-PCR analysis for Plexin-A1 and Neuropilin-1 gene transcripts

RNA was isolated from primary microglia or mouse hippocampi by the SV total RNA isolation system (Promega, Madison, WI, USA). RT of RNA was performed with Super-Script II reverse transcriptase and random primers (Life Technologies). The samples were normalized with β-actin amplification for semiquantification. The primers used for PCR amplification were: Plexin-A1 forward, 5′-GTGTGTGGATAGCCATCA-3′ and reverse, 5′-CCAGCCTCTCGAACACT-3′; Neuropilin-1 forward, 5′-GGCCTCCTGCGATTCGTTACTGCT-3′ and reverse, 5′-CTTAGCCTTGCGCTTGCTGTCATC-3′; and Sema3A forward, 5′-ATTGAATTCAACTATGCAAACGGAAA GAA-3′ and reverse, 5′-TAAGCGGCCGCGACACTTCTG GGTGCCCGCT-3′. Control primers used were: β-actin forward, 5′-GGGACGACATGGAGAAGATC-3′ and reverse, 5′-AGGTCTTTACGGATGTCAACG-3′. All the primers were annealed at 63ºC, and 35 cycles of amplification were performed.

### Immunohistochemistry

Mice were anesthetized by intraperitoneal injection of pentobarbital sodium (0.648 mg/10 g body weight; Kyoritsu Seiyaku Co., Tokyo, Japan) and perfused intracardially with 4% paraformaldehyde. The brain excised from each mouse was fixed in 4% paraformaldehyde, and brain injury was estimated based on the results of hematoxylin and eosin (H&E) staining and immunohistochemistry in consecutive frozen sections of 10 μm prepared from the mouse brain 18 h after ICV injection of the mice. The sections were immunolabeled with anti-Iba-1 antibody (Wako, Osaka, Japan). Microglia were detected by Iba-1 immunostaining, which recognizes both resting and activated microglia. Concurrently, DAPI was used to identify nuclei in the final visualization. Sections incubated in the absence of primary antibody were used as negative controls. To determine the number of Iba-1-positive microglia in the entire hippocampus, three representative images were taken at ×20 magnification in the dentate gyrus and the CA1 and CA3 regions. Using Image J software (National Institutes of Health, Bethesda, MD, USA), a threshold for positive staining was determined for each image in order to include all cell bodies and processes but exclude background staining. The number of Iba-1-positive microglia within the threshold range was manually counted and the average number was calculated for all the representative images. Infiltrated neutrophils were detected by dichloroacetate esterase staining (Muto Pure Chemicals Co., Ltd., Tokyo, Japan). The number of neutrophils that infiltrated into the cerebral cortex following the administration of saline or LPS to the left lateral ventricle was counted at the bregma level of the left hemisphere. Neutrophils found in the meninges were not considered. H&E staining was performed on consecutive 10 μm frozen sections. Left lateral ventricles were sized using Image J software and calculated as a percentage of the total area of the left hemisphere.

### Western blotting

For western blotting analysis, tissue extracts were prepared by homogenizing mouse hippocampus tissue in T-PER Tissue Protein Extraction Reagent (Thermo Scientific Inc., Waltham, MA, USA) containing protease inhibitor, α-complete (Roche Applied Science, Penzberg, Germany). Fifteen micrograms of each tissue extract sample were adjusted to give a final solution of 60 mM Tris-HCl (pH 6.8), 2% SDS, 10% glycerol, 0.1% bromophenol blue, and 5% β-mercaptoethanol. The solution was heated at 100ºC for 5 min, electrophoresed through a 10% SDS-polyacrylamide gel, and transferred to polyvinylidine difluoride membranes (Amersham Pharmacia Biotech, Buckinghamshire, UK). Plexin-A1, Neuropilin-1, COX-2, iNOS, IL-1β, TNF-α and β-actin were detected by their respective antibodies using an enhanced chemiluminescence western blot detection system (Amersham Pharmacia Biotech) according to the manufacturer’s instructions. Anti-Plexin-A1 antibody (Abcam), anti-Neuropilin-1 antibody (Abcam), anti-COX-2 antibody (Santa Cruz Biotechnology, Inc., Dallas, Texas, USA), anti-iNOS antibody (Merck Millipore, Darmstadt, Germany), anti-IL-1β antibody (Santa Cruz Biotechnology), anti-TNF-α antibody (Santa Cruz Biotechnology), and anti-β-actin antibody (Cell Signaling Technology, Danvers, MA, USA) were used.

### ICV LPS injection

Ultra-pure LPS (*Escherichia coli* serotype 055:B5; Sigma, St. Louis, MO, USA) was dissolved in sterile saline at a concentration of 5 mg/ml. Mice were anesthetized by intraperitoneal injection of pentobarbital sodium (0.648 mg/10 g body weight) and placed into a rodent stereotaxic frame (David Kopf Instruments, Tujunga, CA, USA). The scalp was shaved and a burr hole was drilled 0.5 mm caudal to the bregma and 1.0 mm lateral to the midline. LPS (200 μg/kg) was injected via a Hamilton microsyringe (Hamilton Co., Reno, NV, USA) into the ventricle over a 5-min period. Sham animals received an isovolumetric ICV injection of saline.

### NO assay and cell viability assay

To investigate the effect of Sema3A on the microglial production of NO, the nitrite content was measured with Griess reagent (1% sulfanilamide/0.1% N-(1-naphthyl)-ethylenediamine dihydrochloride in 5% phosphoric acid; Sigma) according to the manufacturer’s instructions. Primary microglia were seeded on a 96-well plate at 2×10^4^ cells/well, and incubated overnight in a CO_2_ incubator at 37ºC. Eighteen hours after stimulation of the primary microglia with LPS and Sema3A or control IgG, 50 μl of the culture supernatant were mixed with 50 μl of Griess reagent and incubated for 15 min. Absorbance values were measured at 540 nm in a plate reader, and fresh Dulbecco’s modified Eagle’s medium served as a blank in all the experiments. The NO concentration was calculated with reference to the nitrite standard curve. To analyze cell viability of the primary microglia subjected to NO assay, 5 μl of MTT (5 mg/ml, Sigma, Tokyo, Japan) was added to the primary microglia and incubated for 4 h at 37ºC. Formazan, a product of the MTT tetrazolium ring that was precipitated by the action of mitochondrial dehydrogenases, was solubilized with 0.1 N HCl in anhydrous isopropanol containing 10% Triton X-100 and quantified spectrophotometrically at 595 nm for the measurement of cell viability.

### Statistical analysis

Data are presented as the means ± standard error of mean (SEM). Comparisons between WT and Plexin-A1−/− mice were performed with the Student’s t-test or one-way analysis of variance followed by post-hoc analysis. Statistical significance was established at a level of p<0.05.

## Results

### Plexin-A1 is expressed in mouse microglia

**Figure 1:**
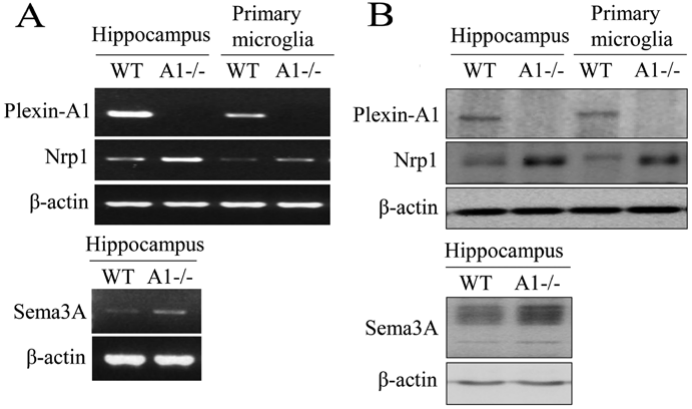
Mouse microglia express both Plexin-A1 and Neuropilin-1. (A) RT-PCR detects mRNAs of Plexin-A1, Neuropilin-1, and Sema3A in mouse hippocampus, and Plexin-A1 and Neuropilin-1 in primary microglia. The method detects no Plexin-A1 mRNA in Plexin-A1-deficient (−/−) hippocampus or Plexin-A1−/− primary microglia. (B) Western blotting detects Plexin-A1, Neuropilin-1, and Sema3A protein in mouse hippocampus, and Plexin-A1 and Neuropilin-1 protein in primary microglia. Immunoblotting detects no Plexin-A1 protein in the Plexin-A1−/− hippocampus or Plexin-A1−/− primary microglia. WT, wild-type; A1−/−, Plexin-A1−/− hippocampus or Plexin-A1−/− primary microglia; Nrp1, Neuropilin-1; RT-PCR, reverse transcriptase polymerase chain reaction.

**Figure 2:**
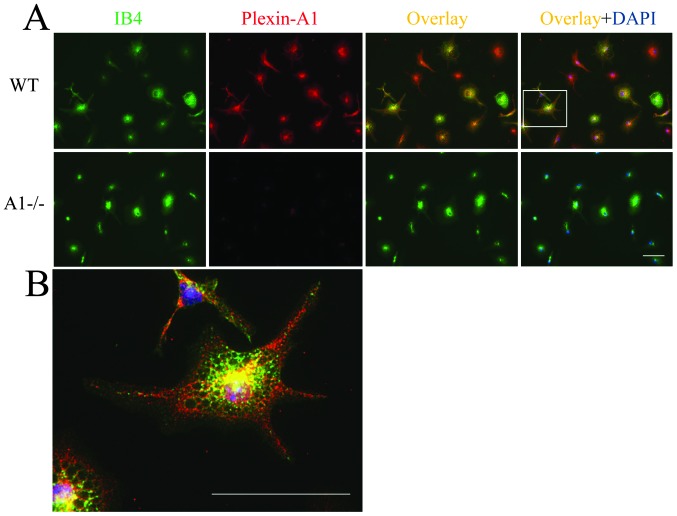
Most primary microglia express Plexin-A1. (A) Cultured microglia double-labeled with specific antibodies against Plexin-A1 (red fluorescence) and specific stain with isolectin IB4 for microglia identification (green fluorescence) show that 98.6±2.3% (mean ± SEM) of WT microglial cells express Plexin-A1. Bar, 50 μm. (B) Overlaid image of WT microglia labeled with anti-Plexin-A1 antibodies and isolectin IB4 is shown at a higher magnification of the selected area in (A). Bar, 50 μm. WT, wild-type primary microglia; A1−/−, Plexin-A1−/− primary microglia; IB4, isolectin IB4; DAPI, DAPI nuclear staining.

**Figure 3:**
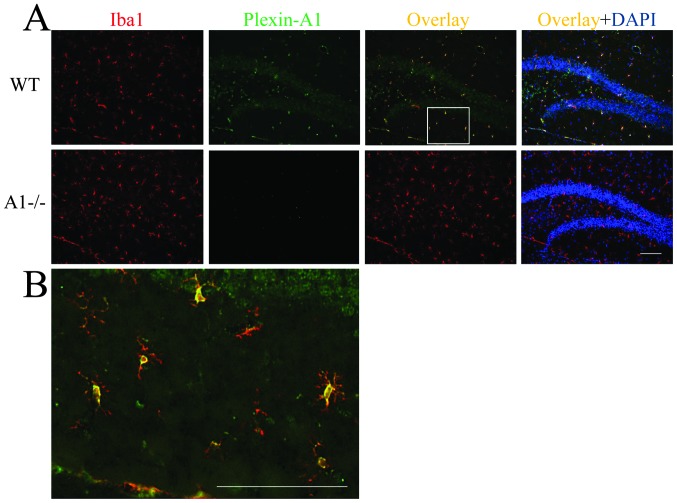
Plexin-A1 is expressed in Iba-1-positive microglia in mouse hippocampus. (A) Plexin-A1-positive immunoreactivity shows a similar distribution pattern with the positive immunostaining for microglial marker Iba-1. (B) Overlaid image of Iba-1 and Plexin-A1 immunofluorescence in WT hippocampus is shown at a higher magnification of the selected area in (A). Bars, 100 μm. A1−/−, Plexin-A1−/− mouse hippocampus; DAPI, DAPI nuclear staining.

Primary microglia were isolated and purified from postnatal mouse brain tissue. For all microglial cultures, purity as determined by lectin cytochemistry analysis was >95%. RNA was prepared from hippocampi and primary microglia and analyzed by RT-PCR. As a result, gene transcripts for Plexin-A1 and Neuropilin-1 were detected in the hippocampus and in primary microglia (Fig. 1A). RT-PCR also detected the expression of Sema3A mRNA in the mouse hippocampus (Fig. 1A). To confirm the expression of Plexin-A1 protein in mouse microglia, western blotting was performed using protein extracts from WT and PlexinA1−/− primary microglia (Fig. 1B). The analysis detected Plexin-A1 protein in WT, but not in Plexin-A1−/− microglia (Fig. 1B). Western blotting also detected Sema3A protein, which in mouse hippocampus is a ligand of Plexin-A1 (Fig. 1B). Expression of Plexin-A1 was detected in primary microglia by double labeling with lectin staining for microglia identification and by Plexin-A1 immunocytochemistry (Fig. 2A and B). In total, 98.6±2.3% (mean ± SEM) of the primary microglia exhibited positive staining for Plexin-A1. To examine the localization of Plexin-A1 in mouse brain, immunohistochemical analyses were performed on hippocampi of WT and Plexin-A1−/− mice. The antibodies against Plexin-A1 detected Plexin-A1 in the microglia in WT (Fig. 3A and B), but not Plexin-A1−/− mice (Fig. 3A).

### Deletion of Plexin-A1 modulates microglial activation status in the LPS response and reduces neutrophil infiltration into the brain

**Figure 4:**
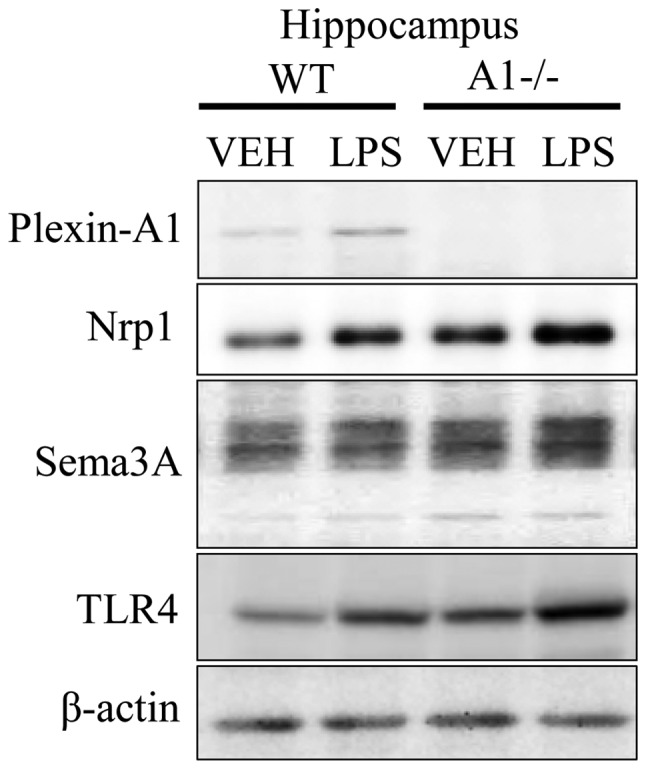
Expression of Plexin-A1, Neuropilin-1, Sema3A, and TLR4 in hippocampus of WT and Plexin-A1−/− mice administered lipopolysaccharide. WT or Plexin-A1−/− mice were subjected to lateral ventricular injection with saline or lipopolysaccharide (LPS; 200 μg/kg). Western blotting detected Plexin-A1, Neuropilin-1, Sema3A, and TLR4 in the mouse hippocampus in all experimental conditions except for Plexin-A1 in the Plexin-A1−/− hippocampus. Nrp1, Neuropilin-1; TLR4, Toll-like receptor 4; VEH, vehicle (saline); LPS, lipopolysaccharide; WT, wild-type; A1−/−, Plexin-A1−/−.

**Figure 5:**
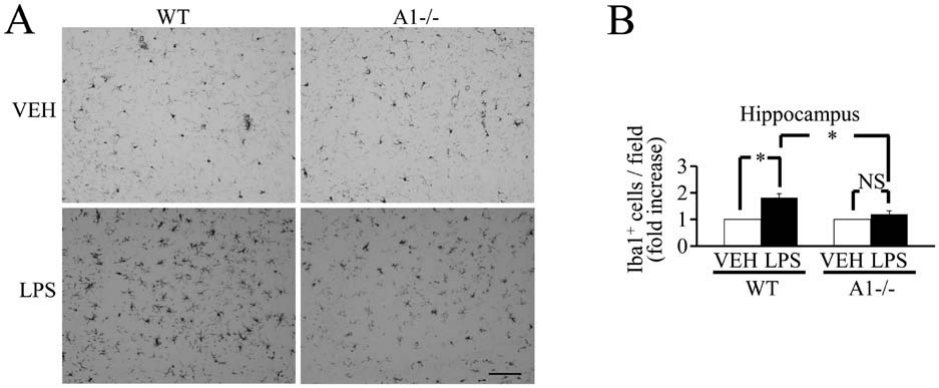
Reduced microglia accumulation in the hippocampus of Plexin-A1−/− mice following lateral ventricular LPS injection. (A) WT mice show an increase in Iba-1-positive microglia in the hippocampus 18 h after LPS injection. Plexin-A1−/− mice do not exhibit any increase of Iba-1-positive microglia in the hippocampus 18 h after LPS injection. Representative image of Iba-1 staining in the CA3 region of hippocampus is shown. Scale bar, 100 μm. (B) Quantification of Iba-1^+^ cells shows a significant increase of microglia accumulation in WT hippocampus 18 h after LPS injection. Quantification of Iba-1^+^ cells also reveals a significant decrease of microglial accumulation 18 h after LPS administration in Plexin-A1−/− hippocampus as compared with WT hippocampus. Bars represent means ± SEM (n=6), ^*^p<0.05. VEH, vehicle (saline); LPS, lipopolysaccharide; WT, wild-type; A1−/−, Plexin-A1−/−.

**Figure 6:**
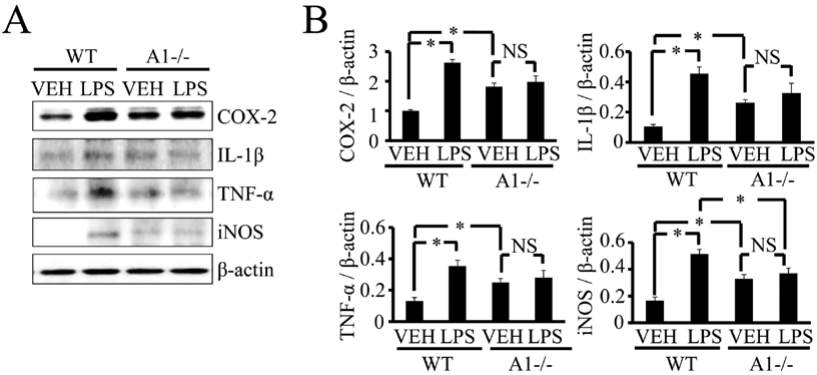
Plexin-A1−/− mice do not show an increase of inflammation-related mediators in the hippocampus after LPS injection. (A) Western blotting shows the increase of inflammation-related mediators in WT hippocampus after LPS stimulation, but not in Plexin-A1−/− hippocampus after LPS administration. (B) Quantification of the immunoblot reveals that the LPS-treated WT group had significantly increased levels of COX-2, IL-1β, TNF-α and iNOS in the hippocampus as compared with the saline-treated WT group. By contrast, the LPS-treated Plexin-A1−/− group does not show any significant increase in levels of COX-2, IL-1β, TNF-α or iNOS in the hippocampus as compared with the saline-treated Plexin-A1−/− group. Results are shown as means ± SEM, ^*^p<0.05. VEH, vehicle (saline); LPS, lipopolysaccharide; WT, wild-type; A1−/−, Plexin-A1−/−.

**Figure 7:**
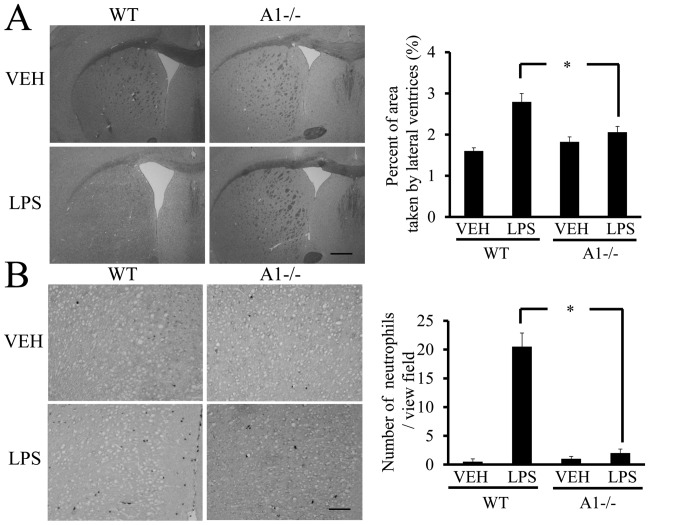
Plexin-A1−/− mice show reduced ventricular enlargement and neutrophil infiltration after LPS injection. (A) LPS injection leads to the enlargement of lateral ventricle in WT mice, but not in Plexin-A1−/− mice. Scale bar is 1,000 μm. Quantification of the area of the lateral ventricle revealed a significant increase in WT mice treated with LPS as compared with saline-treated mice. By contrast, there was no significant increase in lateral ventricular area in the LPS-treated Plexin-A1−/− mice as compared with saline-treated Plexin-A1−/− mice. (B) Esterase staining to detect neutrophil was performed with brain sections from mice injected with saline or LPS. Saline administration to WT mice did not induce any infiltration of neutrophil detected by esterase staining in the cerebral cortex, while WT mice injected with LPS show many infiltrating neutrophils in the cortical area. Esterase staining hardly detects neutrophil in the cerebral cortex even after administration of LPS to Plexin-A1−/− mice. Quantification of neutrophil number shows a significant increase in neutrophil in the cerebral cortex in LPS-treated WT mice as compared with saline-treated WT mice. By contrast, there was no significant increase of neutrophil infiltration into the cerebral cortex in LPS-treated Plexin-A1−/− mice compared with saline-treated control mice. Results are shown as means ± SEM, ^*^p<0.05. VEH, vehicle (saline); LPS, lipopolysaccharide; WT, wild-type; A1−/−, Plexin-A1−/−.

LPS activates microglia through its binding to the LPS receptor, TLR4, on the microglial cell surface (5,6). Plexin-A1−/− microglia may be overactivated in the LPS response due to their inability to use Sema3A to induce apoptosis of activated microglia (28). To test this hypothesis, we compared the microglial responses of WT and Plexin-A1−/− mice following acute administration of LPS to the lateral ventricles. Direct LPS ICV injection led to robust neuroinflammatory responses in the brain, not through activation of peripheral inflammatory cells such as macrophages, but through direct microglial activation (30,31). Immunoblotting studies confirmed the expression of Plexin-A1, Neuropilin-1, Sema3A, and TLR4 in the hippocampus 18 h after ICV injection of saline or LPS (Fig. 4). To elucidate whether the deletion of Plexin-A1 would affect classical microglial activation and the associated inflammatory response, we examined the number of microglia and the expression level of inflammation-related mediators. Of note, 18 h after the ICV injection of LPS, the number of Iba-1-positive cells did not increase significantly in Plexin-A1−/− mice compared with WT mice (Fig. 5), and there was no significant increase in inflammation-related mediators such as COX-2, IL-1β, TNF-α and iNOS (Fig. 6). Thus, levels of brain inflammation after ICV injection of LPS were significantly reduced in Plexin-A1−/− mice as compared to WT mice. During neuroinflammation, changes in vascular permeability lead to the development of brain edema, which in turn results in the enlargement of the lateral ventricles of the brain (32). H&E-stained coronal brain sections revealed that the ratio of the lateral ventricular area to the cerebral hemisphere ipsilateral to the injection site was significantly larger in LPS-injected WT mice as compared to saline-treated mice (Fig. 7A). However, significant enlargement of the lateral ventricle following LPS administration was not observed in Plexin-A1−/− mice (Fig. 7B). Neutrophil leukocytes migrate into the CNS towards various inflammatory stimuli that exacerbate brain tissue damage through the release of inflammation-related mediators and through an increase in vascular permeability (33,34). To examine whether the absence of Plexin-A1 affected neutrophil recruitment, we quantified their infiltration using an esterase stain 18 h after ICV administration of LPS to specifically mark neutrophils. Following administration, the number of esterase-positive cells was significantly fewer in the Plexin-A1−/− cortex as compared to the WT controls (Fig. 7B). Mice administered saline did not show any significant difference based on genotype (Fig. 7B).

### Sema3A increases NO production through Plexin-A1 expression on the microglial cell surface in the LPS response

**Figure 8:**
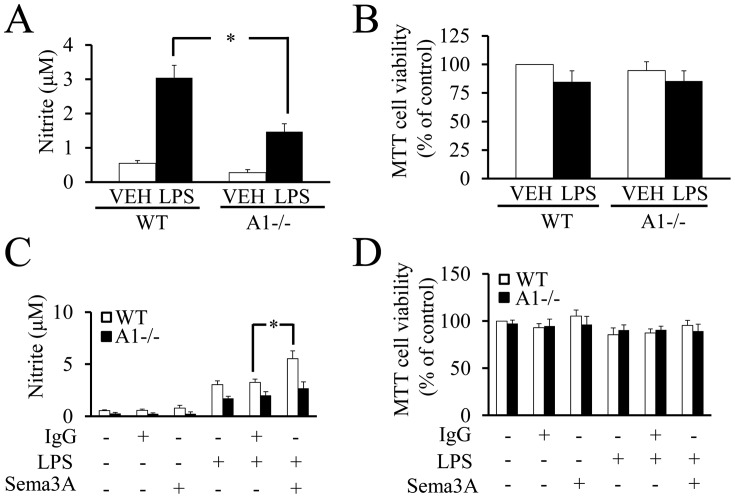
Sema3A increases NO production through Plexin-A1 receptor on microglia in response to LPS. (A) In culture, NO production level is significantly lower in LPS-stimulated Plexin-A1−/− microglia as compared with LPS-treated WT microglia. (B) MTT assay reveals no significant differences in cell viability between each experimental group. (C) Addition of Sema3A and LPS in culture to WT microglia significantly increases NO production as compared with microglia treated with LPS and control IgG. Treatment of Plexin-A1−/− microglia with Sema3A and LPS shows no significant increase of NO production as compared with Plexin-A1−/− microglia treated with LPS and control IgG. (D) MTT assay shows no significant differences between each experimental group. VEH, vehicle; LPS, lipopolysaccharide; WT, wild-type; A1−/−, Plexin-A1−/− microglia.

To directly demonstrate the role of microglial Plexin-A1 in the response to LPS, NO production was measured by the Griess reaction in cultures of WT or Plexin-A1−/− primary microglia stimulated with LPS. The Griess reaction revealed a significant reduction of NO production in Plexin-A1−/− microglia stimulated with LPS as compared with LPS-treated WT microglia (Fig. 8A). There were no significant differences in cell viability among WT and Plexin-A1−/− primary microglia with or without LPS stimulation (Fig. 8B). To examine whether Sema3A enhances the cell response to LPS, NO production was quantified in WT and Plexin-A1−/− primary microglia stimulated with LPS and Sema3A (Fig. 8C). Under stimulation with LPS, the addition of Sema3A to WT microglia exhibited a significant increase of NO production as compared with the addition of control IgG. By contrast, Plexin-A1−/− microglia stimulated with LPS and Sema3A did not exhibit any significant increase in NO production as compared with Plexin-A1−/− microglia stimulated by LPS and control IgG (Fig. 8C). There were no significant differences in cell viability as determined by MTT assay among any of the experimental groups of WT and Plexin-A1−/− microglia (Fig. 8D). Thus, these data suggest a synergistic action of Plexin-A1 activation and TLR4-mediated signaling in microglia.

## Discussion

Results of the present study demonstrated a novel finding regarding the crucial role of Plexin-A1 expressed in mouse microglia, i.e., its activity in enhancing microglial TLR4-mediated signaling in the development of LPS-induced encephalopathy in mice. Although crosstalk between the TLR4 pathway and Plexin-A4-mediated signal was found to play a role in the macrophage response to LPS (35), it remained unclear which plexin signal interacts with the TLR4 pathway to fully activate microglia. Accordingly, to the best of our knowledge, the present study is the first to indicate the importance of synergistic crosstalk between TLR4-mediated signaling and Plexin-A1 activation in the LPS response of mouse microglia.

The present study results demonstrated that the LPS receptor TLR4 and the semaphorin receptor Plexin-A1 acted synergistically in the microglial signal transduction pathway, enhanced microglial activation resulting in neuroinflammation, and contributed to the development of LPS-induced encephalopathy. Microglia activated by LPS are induced to proliferate and produce inflammation-related mediators, suggesting involvement in neuronal injury (6,36). Since microglia activated by neuronal injury in Plexin-A1−/− mice are considered resistant to the induction of apoptosis by the Sema3A secreted from injured neurons (28), Plexin-A1−/− microglia were predicted to react excessively with LPS and facilitate neuroinflammation. Contrary to this prediction, Plexin-A1−/− mice ICV administered LPS, as compared with Plexin-A1−/− mice injected with saline, did not show a significant increase in microglial cell number or in the expression level of inflammation-related mediators (Figs. 5 and 6). These inflammation-related mediators excessively produced by over-activated microglia may act on themselves, thereby inducing microglial cell death (37–40). Since microglia in Plexin-A1−/− mice were significantly decreased compared with the WT in the response to LPS (Fig. 5B), it is possible that overactivation of microglia in Plexin-A1−/− mouse brain induces microglial cell death. However, the TUNEL-positive cell number significantly decreased after LPS administration in the brain of LPS-treated Plexin-A1−/− mice as compared with WT mice (unpublished data). The data indicate a reduced possibility that the decrease of microglial cell number is due to the induction of their cell death by overactivation of microglia. Accordingly, the significant decrease of microglia in Plexin-A1−/− mice after administration of LPS may be derived, not from the overactivation of microglia, but from the inability of Plexin-A1−/− microglia to fully respond to LPS. Activation of intracerebral microglia by LPS administration causes the ventricles to enlarge due to the neuroinflammation-induced brain edema and the increase of leukocyte infiltration migrating towards the TNF-α secreted by activated microglia in the brain (30,41). A significant decrease in the lateral ventricle area and neutrophil invasion in Plexin-A1−/− brain after LPS administration suggests that LPS-dependent activation of Plexin-A1−/− microglia is attenuated (Fig. 7). Furthermore, a significantly lower production of NO after LPS stimulation in Plexin-A1−/− microglia compared with WT microglia demonstrated the essential role of Plexin-A1 for the full enhancement of LPS-induced microglial activation (Fig. 8A). The data suggest that a crosstalk between Plexin-A1 and the TLR4 pathway synergistically enhances the activation of microglia, and thus Plexin-A1-mediated signaling in microglia has an essential role in the development of LPS-induced encephalopathy.

Sema3A may have dual roles in inducing either apoptosis or microglial activation through the Plexin-A1 receptor, depending on the cellular context. A previous study regarding the apoptosis-inducing activity of Sema3A towards activated microglia detected apoptotic microglia with morphological identification of condensed nuclei without using TUNEL or activated caspase-3 staining (28). The expression of activated caspase-3 has been demonstrated to be essential for LPS-dependent microglial activation (42). Therefore, the inhibition of Sema3A-induced cell death by the activated caspase-3 inhibitor reported in a previous study (28) may have an alternative interpretation in which the inhibitor instead suppressed overactivation of microglia. Accordingly, Sema3A may be involved in the crucial autoregulatory mechanism of microglia and in the strengthening of microglial activation through synergistic activation of Plexin-A1-mediated signaling and the TLR4 pathway, but it may also induce apoptosis of excessively activated microglia in order not to kill neurons in close proximity to the over-activated microglia. Our *in vitro* model, however, suggests that Sema3A-induced Plexin-A1 signaling is required for LPS-induced microglial activation, but not for the apoptotic induction of activated microglia (Fig. 8C and D). In the LPS response outside the brain, the signaling of Sema3A through Plexin-A4 (another member of the Plexin-A family) has crosstalk with TLR4-mediated signaling in macrophages, and plays a crucial role in exacerbating cytokine storms (35). Furthermore, Plexin-B1 on microglia bound with the Sema4D ligand has been demonstrated to be necessary for the inflammatory mediator-dependent activation of microglia (43). Our findings further develop the study of the regulatory mechanisms of the Plexin family in the inflammatory response. Therefore, results of the present study suggest that the regulatory mechanism of the semaphorin-Plexin signaling system may be applicable to the treatment of LPS-induced encephalopathy and other psychiatric diseases associated with neuroinflammation.
